# Experimental study on the influence of facial muscle activity on the facial mesostructure bones in rabbits

**DOI:** 10.1016/S1808-8694(15)31377-X

**Published:** 2015-10-17

**Authors:** Andre Ricardo Mateus, José Eduardo Lutaif Dolci, Henrique Olavo de Olival Costa, Flávia Coelho Sousa, Noemi di Biase

**Affiliations:** 1 Master’s degree, otorhinolaryngologist, Hospital Santa Catarina, São Paulo, SP; 2 Doctoral degree, director of the Otorhinolaryngology Department, Faculdade de Ciências Médicas da Santa Casa de São Paulo; 3 Doctoral degree, coordinator of the Otorhinolaryngology Graduate Course Council, Faculdade de Ciências Médicas da Santa Casa de São Paulo; 4 Veterinarian, responsible for the ICAO biotherium, ICAO - Instituto de Ciências Avançadas em Otorrinolaringologia; 5 Doctoral degree, otorhinolaryngologist

**Keywords:** face, facial muscles, facial palsy

## Abstract

Based on the functional matrix concept, scientists developed the hypothesis that soft tissue acting on certain bone pieces determines the process of facial growth. The possibility to modify muscle influence in the phase of facial development, or in postoperative of corrective surgery is of great preventive importance and it should be better investigated, since it could reduce the number and impact of these procedures.

**Study design:**

experimental in rabbits.

**Aim:**

to estimate the relevance of facial muscle activity on facial bones in lab rabbits.

**Materials and Methods:**

37 rabbits of two months of age were studied, divided in a study group and a control group, were followed up for a period of 4 months. The study group animals had their facial nerves cut at the cervical root in one side. The facial mesostructure of the animals was removed in block for later morphometric studies through computer graphics made out of the digital pictures of the specimens. Results were submitted for comparative statistical analysis.

**Conclusion:**

The lack of muscle activity in half of the face produces an ipsilateral shift of the facial mesostructure in developing rabbits.

## INTRODUCTION

Studies on craniomaxillofacial embryology and growth have developed fundamental concepts for understanding and treating congenital and acquired facial deformities.^1^

Various theories have tried to explain facial growth based on the fact that the face grows forwards and downwards, and that each bone component has ossification sites with different onset and formation rates.^2^

The human face develops in an anterior and inferior projection.^3^

According to the nasal septum theory, endochondral growth of this cartilage causes a sliding physical force over the nasal crest that displaces the maxilla forwards and downwards, resulting in this form of growth.^3^

The functional matrix theory states that soft tissues acting on the various bony parts composing the face are the determining factor for its anterior and inferior growth pattern.^4^

Compression of the periosteum decreases the amount of blood flowing locally, which causes activation of osteoclasts and a resulting underlying bone resorption. Inversely, traction forces on the periosteum causes osteoblasts to be activated, with resulting bone apposition in an effort to keep the bone fixed to its coating membrane.^5^

The forces resulting from cartilage development in the nasal septum^3^ are useful only in the intrauterine growing and development phases, and appear to lose importance relative to the extrinsic forces exerted by soft tissues after birth.^4^, ^5^, ^6^

All external remodeling on a bone part is invariably accompanied by an inverse physiological phenomenon in the corresponding internal site. This assures that the bone component is remodeled, rather than only thickened.^6^

**Figure 1:**
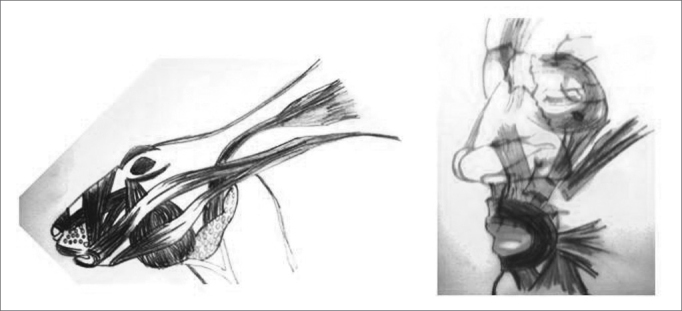
Comparative schematic representation of facial mesostructure muscles in humans and in rabbits (Mateus, 2007)

Figure 1 shows the anterior and inferior arrangement of the muscle groups acting on the facial mesostructure of rabbits and humans.

The possibility arises that differences in the forces produced by muscle contraction in each half of the face may induce facial asymmetry,^7^ based on the functional matrix theory^4^, ^5^, ^6^ and its probably influence on the anterior and inferior projection of the face during growth and development.^3^, ^5^

**Figure 2:**
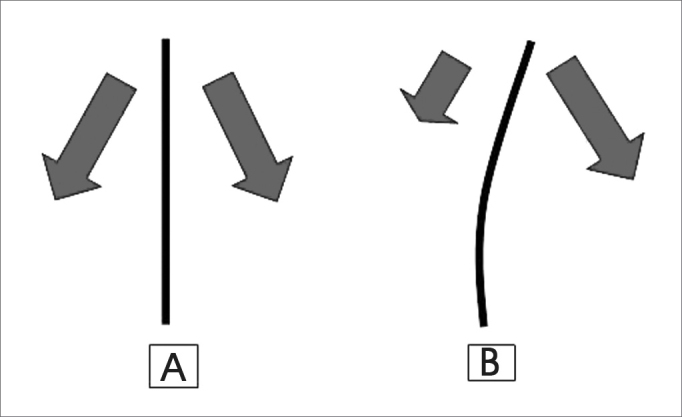
Arrows show the force vectors produced by muscles operating on both halfs of the face. (A) symmetrically, and (B) asymmetrically (Mateus, 2007)

Figure 2 shows the force vectors of muscle activity in each half of the face.

The susceptibility of the skeletal unit of the nasomaxillary complex to these forces - due to its location along the median line and the center of the face - would be important for facial reconstruction surgery and especially for rhinology. We thus decided to undertake an experimental study to estimate the influence of the facial muscle activity on the facial mesostructural skeleton.

## OBJECTIVE

The purpose of this study was to estimate the influence of facial muscle activity on the growth and development of the facial mesostructural skeleton in rabbits.

## MATERIAL AND METHOD

The Research Ethics Committee for animal experimentation assessed and approved this study (protocol number 137).

Animal vivisection was done according to the ethical principles for experimentation in the Código Brasileiro de Experimentação em Animais - COBEA (Animal Experimentation Brazilian Code) and according to the Federal Law nº 6638 of 8 May 1979.

A pre-study was done of five rabbits to standardize the surgical technique used in this study. One of these rabbits was dissected for an anatomical study.^8^, ^9^, ^10^

Thirty-seven New Zealand rabbits aged two months (phase of most significant growth of these animals), with no gender preference, in good nutritional status, weighing between 1,300 grams and 1,670 grams, were selected. The activity of perinasal muscles was checked bilaterally in all animals by observing the movement of nasal vibrissae.^11^

The rabbit sample was randomly divided into two groups, as follows:

Control consisting of 19 rabbits that developed during the study period with no interference.
Operated consisting of 18 rabbits that underwent surgery for causing unilateral peripheral facial paralysis in the perinasal muscle group. The operated side was defined randomly; 13 rabbits were operated on the right, and five rabbits were operated on the left.

Vivisection was done after the animals adapted to the biotherium. A veterinarian with experience in dealing with laboratory animals guided the procedure.

The same surgeon (the researcher) carried out all procedures, using a standardized technique for resecting a segment of the facial nerve from the stylomastoid foramen coursing towards the perinasal muscle group of one half of the face.

Rabbits in the study group were subjected to deep hypnosis with intramuscular 0.5 ml/Kg ZoletilR (125 mg/5 ml tiletamine chloridrate and 125mg/5ml zolazepam chloridrate) and 0.3 ml/Kg NilperidolR (50 mcg/ml fentanyl citrate and 2.5 mg/ml droperidol).

Tracheal intubation was not required in any animal; all were ventilated spontaneously during surgery.

Retroauricular trichotomy was done with an electrical device for removing animal fur.

Each animal was placed on an operating table in lateral decubitus with the head extended; the procedure was undertaken with aseptic material, staring with antisepsis of the area to be operated with topical PovidineR.

A Welch-AllynR 15-Watt halogen light photophore provided artificial illumination. Topical anesthesia with 2% XylocaineR (lidocaine chloridrate) was done along the incision site in addition to intraoperative analgesia.

Antibiotic prophylaxis (penicillin G procaine - 0.1 ml/Kg) was given to all rabbits. Dipyrone was used for analgesia at the immediate postoperative period and every 24 hours.

Rabbits weighed between 3,600 and 4,050 grams at age six months (onset of adult life). An intracardiac lethal dose (2 ml) of 19.1% KCl (potassium chloride) following deep hypnosis produced with the same drugs and doses used during the surgical procedure.

Rabbits were decapitated and dissected for obtaining the craniofacial skeleton. Each specimen was kept in 70% formaldehyde in plastic containers and refrigerated at 4 degrees Celsius.

Standard marking for assessing facial mesostructural symmetry was made on specimens with 3 mm diameter black spherical tip needles.

Standard points for marking were located on the inferior face of the specimen, since this is the area with the largest number of visible surface anatomical landmarks. It also allowed a better assessment of facial mesostructural symmetry, since the marking points were easy to identify and there were more possibilities for establishing topographic relationships with other anatomical structures on this view.

The points that were chosen were:

- intersection point between major and minor incisors along the median line;
- intersection point of the transverse and median palatine sutures;
- anterior portion of both first molar teeth.

Each duly marked specimen was photographed following a standard method. A Sony DSC-P8 Cyber-shot digital camera (3.2 megapixel, 3x optic zoom) was used for taking the pictures. The camera was placed on a tripod, 50 centimeters from a flat opaque background surface colored green onto which each specimen was placed. Specimens were aligned by orienting the longitudinal palatine groove along the same direction as a white line drawn on the background parallel to the tripod. The photographic equipment was placed under the sunlight that entered through a window of a room with white walls and ceiling for photography.

Pictures were saved in a Toshiba Satellite laptop (Intel processor, 30 Gbytes HD, 256 Kbytes RAM, running Windows XPR).

A geometrical analysis of markings was made on the pictures using the CorelDRAWR 12 graphic computer software. An imaginary line was traced linking the mark on the intersection of palatine sutures to the mark on the corresponding orifice to the central medullary canal, following a median orientation overlapping the tracing of the longitudinal palatine suture. The lateral deviation angle was measured using this imaginary line as a parameter, and starting from the mark of the intersection of palatine sutures, tracing a new imaginary line linking this point to the intersection of incisor teeth. The graphic computer software measured the angle between these imaginary lines; it represented the degree of lateral deviation of the anterior extremity of the facial mesostructure.

Data and the statistical analysis were tabulated in charts and tables for comparing both groups (control and study). The T test and F test were used.

## RESULTS

### Presentation of data

Table 1 shows that we found no deviation equal to or above 4 degrees in the control group; in the study group (operated animals), 8 of 18 animals (44.44%) had that level of deviation.

**Table 1:** in degrees of lateral deviation of the facial mesostructural skeleton.

Angle	Control	%	Operated	%
0	4	21,05	5	27,77
1	1	5,2	1	5,55
2	9	47,36	3	16,66
3	5	26,31	1	5,55
4 ou	0	0	8	44,44

TOTAL	19	100	18	100

Taking into account a lateral deviation equal to or above 3 degrees, we found that about 26% of the control group and nearly half of the operated group were included (Table 1).

**Figure 3:**
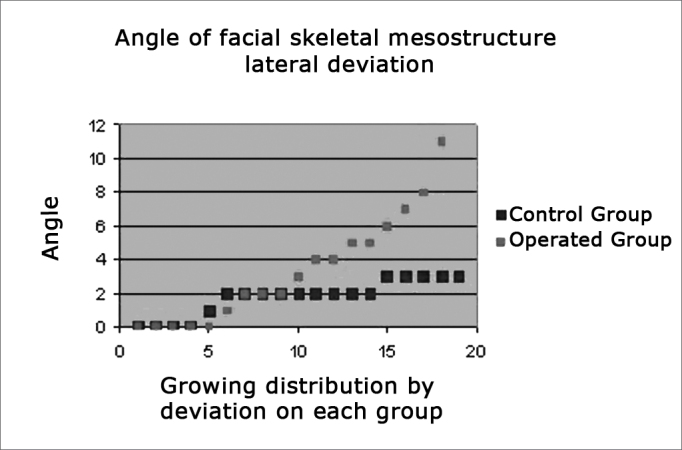
The chart shows lateral deviations of the facial mesostructure in both study groups

Figure 3 shows in chart form where the X-value axis shows the angle of the lateral deviation of the facial mesostructural skeleton; the Y-value axis shows the animals distributed within each group in growing order according to the degree of deviation.

We found a more homogeneous distribution in the control group (shown in blue), with lateral deviation angles around 2 degrees. In the operated group (shown in red) we found many animals with deviations of 4 degrees or more, mostly around 6 degrees; the highest deviation was 11 degrees.

**Figure 4:**
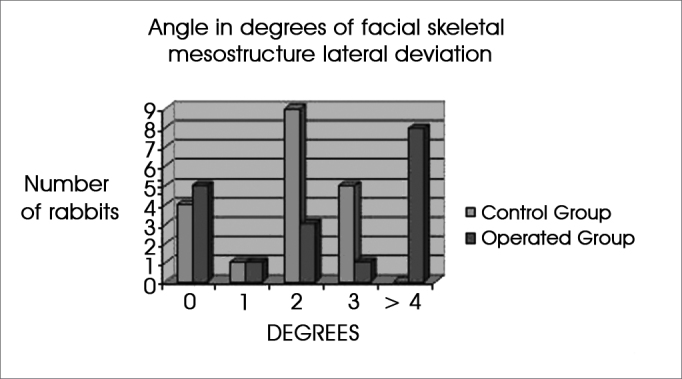
The chart shows the number of rabbits that demonstrated each degree of lateral deviation of the facial mesostructure, in both study groups

Figure 4 shows in height of columns that most of the control animals (shown in blue) had 2 degrees of lateral deviation. In the study or operated group (shown in red), most of the animals had deviations equal to 4 degrees or more; no control group animal was within this class of deviation (shown in the chart as the height of Y-values).

Table 2 shows that the deviation in all control group animals with any degree of lateral deviation angle in the facial mesostructural skeleton was to the right.

**Table 2:** Side of lateral deviation of the facial structure in each group.

	OPERATED	CONTROLS
RIGHT	8	15
LEFT	5	0
NONE	5	4

TOTAL	18	19

Table 3 shows that the side with no muscle activity was that to which the anterior end of the facial mesostructural skeleton moved in operated rabbits.

**Table 3:** Comparison between the side in which muscle activity was absent and the side that presented deviation in operated rabbits.

	OPERATED SIDE	NO DEVIATION	SAME SIDE	OPPOSITE SIDE
RIGHT	12	4	8	0
LEFT	6	1	5	0

TOTAL	18	5	13	0

### Statistical analysis

A non-paired comparative analysis using the F test resulted in P<0.001; the T test resulted in P=0.05. Both were considered as statistically significant.

## DISCUSSION

The fact that the facial skeleton grows anteriorly and inferiorly brings our attention to the study of the growth and development of the nasomaxillary complex. Understanding that forces produced by structures that fulfill the concepts of the functional matrix theory^3^, ^4^ replace the influence of quadrangular cartilage growth forces^2^, ^5^ provides us with a basis for understanding facial growth after birth. The influence of soft tissues - particularly the facial muscles - on the craniofacial skeleton controls bone resorption and deposition, which sets the shape and symmetry of the facial skeleton.

A description of the physiological phenomenon of external remodeling of a bone part^6^ led us to believe in an ongoing balance between skeletal morphology and the influence of forces produced by its functional matrix, which may stop or establish facial asymmetries. This balance is the basis for our experimental study.

We produced unilateral peripheral facial paralysis in growing and developing rabbits to test the influence of the muscle component of the functional matrix on bone parts of the facial mesostructure by evaluating the symmetry of the facial skeleton.

We chose the rabbit as our model since this animal has a wide variety of facial muscle movements and ample nasal soft tissue movements. Furthermore, the nasomaxillary complex is significantly projected forward in rabbits in its adult skeletal conformation, which facilitated measuring possible asymmetries results from our experiment.

Rabbits were useful as experimental animals for assessing craniofacial growth, the facial musculature and its motor innervation.

Successful use of graphic computation techniques for measuring linear and angular values in computerized cephalometry and photogrammetry led us to develop the method for assessing the nasomaxillary complex facial skeleton symmetry in rabbits in this study.

Our results demonstrated the influence of functional matrix facial muscles on the facial skeletal conformation in rabbits. We were able to attain faster results by using rabbits in the growing and developing phase. Rabbits become adults at age six months; we were thus able to collect our results only four months after starting our experiment.

The evaluation of spontaneous or touch-stimulated vibrissae movement7 was the basis for assessing the functional integrity of the perinasal muscle group for selecting those rabbits that were to be included in the study.

The decision to section the cervical branch of the facial nerve gave us more control of the perinasal muscle group in the rabbits. Although we not necessarily observed signs such as earlobe ptosis (we sectioned the nerve without identifying the early nerve division at the stylomastoid foramen that forms the posterior auricular nerve) or reduced ocular blink reflex on the half of the face in which we produced peripheral facial paralysis,7 our sample showed statistically significant (T and F tests) lateral deviation of the skeletal facial mesostructure.

In our view, the importance of the perinasal facial muscles in forming the nasomaxillary skeleton - described in rabbits in our study - raises the need for taking into account the functional matrix concept as applied to this skeletal unit4 when undertaking esthetic and functional therapy of patients with lateral deviation of the nasal pyramid.

Different from what we imagined, the effect of muscle forces on the active half of the face in rabbits subjected to unilateral facial paralysis in our study was not to exert traction on the anterior extremity of the face to cause lateral deviation towards its side (Figure 2).

As explained by the functional matrix theory,^3^, ^4^ the effect of muscle force on the unoperated facial half resulted in bone deposition under the periosteum of that area; this helped form a mesostructural curvature that was convex towards the side from which traction occurred. Thus we found that the side affected by lack of muscle activity had a concave curvature in all operated animals that presented some degree of facial mesostructural skeletal lateral deviation (Table 2 and 3). This suggest that the facial mesostructural skeleton behaved as a bone structure undergoing remodeling in our study, abiding by the physiological principle of bone resorption in the inverse site relative to the surface on which bone apposition occurs.^6^

Physical therapy, medication or surgical therapy on nasal pyramid muscles may in future be an effective measure to reduce recurrences in operated crocked noses.

The difficulty is deciding on what muscle group should we intervene. These muscles tend to fuse with adjacent muscles and vary in size and strength.^1^ Directly over the nasal septum, unilateral hypotrophy of the septal depressor muscle may contribute to recurrences in operated caudal septal deviations and the resulting lateral deviation of the nose tip. Shortening of the transverse muscle fibers of the nasal muscle might cause recurrences of lateral deviations of the nasal pyramid treated surgically. Hypotrophy of the longitudinal fibers of the upper lip elevator muscle and the nasal alae might cause difficulties for undertaking rhinoseptoplasties in subjects with nasal dorsum deviations.

Guided physical therapy shortly before and soon after surgery - consisting of stretching forces on the nasal pyramid to stimulate muscle fiber stretching - may be done as coadjuvant therapy for treating nasal lateral deviations.

Botulin toxin on the skeletal muscle acting on the nose might at least temporarily reduce the possibility of one muscle group altering the results of surgery for deviated noses, while consolidation of bone structures and cartilage take place.

Myotomy of specific skeletal muscle fibers acting on the nose may inhibit muscle lateral traction forces on the nasal pyramid in operated cases of crocked noses.

## CONCLUSION

Absence of muscle activity in one of the half of the face results in lateral deviation of the facial mesostructural skeleton to the same side in growing and developing rabbits.
